# Genetics of Congenital Heart Disease

**DOI:** 10.3390/biom9120879

**Published:** 2019-12-16

**Authors:** Kylia Williams, Jason Carson, Cecilia Lo

**Affiliations:** Department of Developmental Biology, University of Pittsburgh School of Medicine, Pittsburgh, PA 15201, USA; kyw13@pitt.edu (K.W.); jcc141@pitt.edu (J.C.)

**Keywords:** congenital heart disease, heart development, transcription factors, signaling pathways, chromatin modification, ciliary function

## Abstract

Congenital heart disease (CHD) is one of the most common birth defects. Studies in animal models and humans have indicated a genetic etiology for CHD. About 400 genes have been implicated in CHD, encompassing transcription factors, cell signaling molecules, and structural proteins that are important for heart development. Recent studies have shown genes encoding chromatin modifiers, cilia related proteins, and cilia-transduced cell signaling pathways play important roles in CHD pathogenesis. Elucidating the genetic etiology of CHD will help improve diagnosis and the development of new therapies to improve patient outcomes.

## 1. Introduction

Congenital heart disease (CHD) is a form of birth defect that affects about 1% of infants born each year. Disturbances in heart development result in a variety of defects, and while CHD can be caused by environmental exposures to teratogens [1,2], a genetic underpinning for CHD is strongly supported by the observation of a high recurrence risk and familial forms of the disease, as well as the well-described association of CHD with chromosomal anomalies [3].

It is estimated that about 400 genes are associated with CHD pathogenesis. Mutations in genes encoding transcription factors, cell signaling transducers, and chromatin modifiers can interfere with cell type specification, differentiation, and patterning important in heart development causing perturbations in heart structure and function. As many of the proteins encoded by these genes work synergistically or are connected by functional networks, this suggests a broad interacting network may be associated with disease [4,5]. However, ~60% of CHD cases remain unexplained, as studies into the genetic etiology of CHD have been confounded by the genetic diversity of human subjects [6]. Also confounding genetic inquiry is the genetic heterogeneity associated with CHD. Together this has resulted in variable expressivity where subjects with the same variants may exhibit different phenotypes, or variable penetrance where some individuals with a known pathogenic variant may have no disease. As a result, CHD largely has a non-Mendelian inheritance patterns and is best described as mediated by complex genetics.

There have been several studies utilizing targeted whole-exome or whole-genome sequencing to investigate the genetic basis for CHD. In trio studies, the proband is sequenced along with unaffected parents to identify pathogenic variants that may have arisen de novo. In familial studies, multiple members of a family are phenotyped and sequenced to identify variants that are inherited in diseased family members. In cohort studies, a large number of unrelated cases and healthy control samples undergo sequencing to determine if any single gene or set of genes is enriched for variants in the disease samples. Studies of de novo and rare inherited variants have revealed a higher burden of mutation in variants predicted to be damaging in genes associated with CHD, highly expressed in the heart, or involved in heart development [6]. Among these variants, there is a surprising number of ciliary genes and genes encoding chromatin modifiers. There is also a high burden of rare copy number variants in CHD patients, which is likely driven by syndromic cases [7].

Our understanding of the genetic causes of CHD has also benefited from studies in mouse models. Inbred mice provide an ideal context to conduct genetic analysis, and importantly, mice have the same four-chamber cardiac anatomy as humans that are susceptible to CHD pathogenesis [8]. Given this, as well as the rapid advances in reverse genetics for generating gene knockouts, knock-ins, and point mutations, mice have become the model of choice to interrogate the genetic causes of CHD. These have allowed for the rapid verification of CHD candidate genes with disease modeling in vivo, along with in vitro cell and tissue culture studies. The recent use of patient-derived induced pluripotent stem cells (iPSCs) have become especially valuable for mechanistic studies. Using mice, it is also possible to interrogate the genetic etiology of CHD using forward genetics with chemical mutagenesis. Using such forward genetic screening methods with ethylnitrosourea (ENU) mutagenesis, our laboratory has identified over 100 genes causing CHD [5]. Forward genetic screens are advantageous in that they are entirely phenotype-driven, so there is no a priori gene bias, allowing the possibility for discovery of new biology.

In combination, these human and animal studies have helped to elucidate the genetic etiology of CHD and the underlying molecular mechanisms driving disease. Below, we will first briefly describe the classification of CHD and developmental processes orchestrating heart development and formation of the mammalian heart. Next, the major transcription factors and signaling pathways associated with CHD will be briefly reviewed, with a focus on genes known to be causal of CHD from mouse and human studies. Lastly, we will touch on the role of chromatin modifiers, cilia, cilia-transduced cell signaling, and maternal factors in CHD pathogenesis.

## 2. Congenital Heart Disease Classification and Prevalence

CHD encompasses a variety of cardiac defects that are commonly grouped based on the nature of the structural heart defect [9,10], resulting blood flow patterns [11], observed familial recurrence risks [12,13,14], and shared susceptibility genes [12]. Phenotypes are often sorted into major categories such as right-sided lesions, left-sided lesions, conotruncal defects, laterality defects, and isolated septal defects. Right-sided lesions include hypoplastic right heart syndrome (HRHS), Ebstein’s anomaly, and pulmonary artery atresia. Left-sided lesions include bicuspid aortic valve (BAV), aortic stenosis, coarctation of the aorta (CoA), and hypoplastic left heart syndrome (HLHS). Conotruncal defects include tetralogy of Fallot (TOF), pulmonary atresia, truncus arteriosus, and double outlet right ventricle (DORV) except those with malposed vessels or HLHS. Laterality defects include heterotaxy (HTX), atrioventricular septal defects (AVSD), anomalous pulmonary venous return (APVR), transposition of the great arteries (TGA), malposed vessels, dextrocardia, and situs inversus totalis (SIT). Isolated septal defects include atrial septal defects (ASD) and ventricular septal defects (VSD) [9]. A meta-analysis of global birth prevalence of CHD showed that the ‘mild lesions’ ASD, VSD, and patent ductus arteriosus (PDA) account for 57.9% of CHD burden [15]. The prevalence of these mild lesions, as well as severe complex CHD, has risen ~10% every 5 years since 1970 [15]. CHD associated with chromosomal abnormalities represents ~8%–10% of all CHD [3] and is believed to have a separate genetic etiology from non-syndromic disease, with a greater proportion driven by protein truncating and missense de novo mutation [16].

## 3. Developmental Processes in Formation of the Four-Chambered Heart

The heart is one of the first organs to develop during embryogenesis. In response to endoderm- and ectoderm-derived Bmp, Fgf, and Wnt signaling in the early mouse embryo, embryonic precursors derived from the mesoderm give rise to cardiac progenitors in the cardiac crescent [17]. These cells migrate and fuse along the midline, generating the linear heart tube. This is followed by looping of the heart tube, with the outer curvature of the looped heart tube forming the future ventricles, while the venous pole becomes the atrial appendages [18]. In parallel, the conotruncal outflow undergoes septation to generate the aortic and pulmonary arteries. Neural crest cells migrating into the heart play a critical role in regulating outflow septation. Correct alignment of the outflows such that there is proper connection of the aorta with the left ventricle (LV) and pulmonary artery with the right ventricle (RV) is mediated by wedging of the outflows between the cardiac cushions such that there is “mitral to aortic valve continuity” [19]. Formation of the cardiac valves is mediated via epithelial-to-mesenchymal transition (EMT) of endocardial cells that form swellings known as the endocardial cushions. The cushions serve as primitive valves early in development, but later remodel to form the mature thin valve leaflets [18]. The atrioventricular (AV) valves are formed from superior and inferior atrioventricular cushions that later fuse with the growing muscular septa between the atria and ventricles. The outflow tract cushions give rise to semilunar valves of the aorta and pulmonary trunk [20].

Lineage tracing experiments have provided significant insights into the developmental etiology of different structures of the four-chamber heart [18]. While the linear heart tube is comprised of cells from first heart field (FHF) that will give rises to the future LV and part of the atria, cells from the second heart field (SHF) migrate into either pole of the linear heart tube, giving rise to the OFT, RV, and also part of the atria. When the linear heart tube undergoes looping, bilateral symmetry is broken with the direction of looping reflecting the left–right body axis. This left–right patterning is of critical importance since the heart is one of the most left–right asymmetric organs in the body. This asymmetry is required for efficient oxygenation of blood, establishing circulation from the right side of the heart to the lungs for oxygenation, while the left side pumps oxygenated blood systemically throughout the body. Thus, when left–right patterning is disrupted, such as with randomization of visceral organ situs in HTX, there is invariably complex CHD.

## 4. Role of Transcription Factors

A combination of clinical studies and studies using mouse models have allowed the identification of transcription factors and cofactors involved in CHD and uncovered their roles in CHD pathogenesis (Table 1). The further identification of novel variants and CNVs has emerged from large cohort studies [21,22,23]. Transcription factors in CHD patients are also observed to be enriched for de novo and loss of function mutations [10]. Proteins with such deleterious mutations displayed changes in transcriptional or synergistic activity, which can interfere with expression of downstream targets, causing the perturbation of cell type specification, and differentiation [21].

### 4.1. NKX2-5

*NKX2-5* encodes a homeobox transcription factor that plays an important role in heart development. It is expressed at the earliest stages of cardiogenesis, regulating cardiomyocyte differentiation and proliferation [24]. *NKX2-5* mutations were first identified to cause AV block and ASD [25,26], but have since been recovered in a wide spectrum of CHD. Moreover, the phenotype and penetrance of *NKX2-5* mutations have been shown to be dependent on genetic background and interaction with other mutations in both mice and humans [22,25,27,28]. Together, these findings have complicated investigations into mechanisms by which NKX2-5 mutations cause CHD. In vitro mouse modeling of a heterozygous mutation in *Nkx2-5* associated with AV block and ASD showed reduced NKX2-5 nuclear import, downregulation of BMP and Notch signaling, and ultimately dysregulation of genes involved in early cardiomyocyte differentiation and function and reduced cardiomyogenesis [29].

### 4.2. GATA Family

GATA4, 5, and 6 are zinc finger transcription factors that have been shown to be expressed in the developing heart and have roles in cardiogenesis [30]. Mutations in *GATA4* that decrease transcriptional activity have been associated with BAV and VSD [31]. Mutations in genes that regulate *GATA4*, such as *NEXN*, have also been associated with CHD [32]. *Gata4* has been shown to be required by Hh-responsive progenitors within the SHF involved in OFT development, with a heterozygous *Gata4* mutation shown to cause VSD and OFT defects in mice, including DORV and AVSD [33]. Noncoding variants in *GATA4* have also been associated with BAV, illustrating the importance of further research into noncoding and regulatory regions of the genome [34]. Heterozygous mutations in *GATA6* also have been identified in CHD patients. Studies in mice showed *Gata6* mutations can cause severe OFT defects through disruption of Sema3c and Plxna2 expression [35,36]. Mice that are double homozygous knockouts for *Gata4*/*Gata6* exhibit acardia and only generate SHF progenitor cells [33]. Mutations in *GATA5* have only more recently begin to be explored as a cause for CHD. Rare sequence variants have been reported in patients with TOF, VSD, familial atrial fibrillation, and BAV [37], and loss of *Gata5* results in BAV in mice [38].

### 4.3. T-Box Family

The TBX transcription factors are expressed throughout the developing heart and play a key role in regulating cardiomyocyte identity [18]. Mutations in *TBX1*, which is expressed in outflow tract precursors, have been found in patients with DiGeorge syndrome, which is commonly associated with cardiac defects. Loss of transcriptional targets of *Tbx1*, such as *Wnt5a*, also cause severe hypoplasia of SHF-dependent structures in mice, similar to loss of *Tbx1* [39]. In addition, CNVs affecting *PRODH* and *DGCR6*, which have been reported to affect *TBX1* expression, have been associated with conotruncal defects in DiGeorge patients [40]. TBX5 and TBX20 activate gene expression in the cardiac chambers, TBX2 and TBX3 repress myocardial gene expression in the inflow and outflow tract precursors, and TBX18 is expressed in the venous pole. Deletion of these genes in mice result in a variety of cardiac defects [41]. *TBX5* and *TBX20* both drive chamber formation from FHF progenitors. Mutations in *TBX5* are known to cause Holt–Oram syndrome, which is characterized by heart and upper limb deformities [42]. Studies in mice showed *Tbx5* interacts with both *Gata4* and *Gata6*, such that double heterozygous mutations with *Gata6* result in neonatal lethality, and double heterozygous mutations with *Gata4* result in more severe cardiac malformations and embryonic lethality. Mutations in *TBX20* have also been associated with CHD such as TOF, and knockdown of *Tbx20* in mice suggests that it plays a role in development of the SHF [41].

### 4.4. Forkhead Box Family

Several forkhead box (FOX) transcription factors also play important roles in heart development, with mutations leading to cardiac defects and embryonic lethality [43]. Deletion CNVs of the *FOXF1*, *FOXC2*, and *FOXL1* are associated with CHD, particularly HLHS [44]. Mutations in *FOXC2* are a well-characterized cause of TOF [45]. A mutation in FOXF1 was identified in one patient with AVSD, hypoplastic LV, bicuspid aortic valve, and also intestinal malrotation, indicating disturbance of left–right patterning. Another patient with VACTERL and HTX, was also identified with a mutation in *FOXF1* as well as *ZIC3*, both of which regulate the specification of laterality [46,47]. *FOXA2* has been shown to regulate *TBX1* transcription and development of the outflow tract [43]. Mutations in *FOXH1*, a downstream target of the Nodal pathway signaling, have been identified in patients with VSD, TGA, and laterality defects [48,49]. Mutations in *Foxj1*, which is a regulator of ciliogenesis, were identified to cause complex CHD with HTX in a large-scale mouse mutagenesis screen [5].

### 4.5. Nuclear Receptor Family

A de novo mutation in the DNA binding domain of *NR1D2,* a nuclear receptor transcriptional repressor that acts in a heme-dependent manner, has been identified in a cohort of patients with AVSD [50]. It was shown to change transcriptional activity, and knockout mice were shown to have cardiovascular malformations. Another nuclear receptor, *NR2F2* encodes a pleiotropic transcription factor shown to be required for normal development of the atria, coronary vessels, and aorta [51]. In a mouse model, cardiomyocyte-specific knockout of *Nr2f2* resulted in ventricularized atria. A mutation in *NR2F2* was found to segregate with disease in a family with DORV and VSD and absent in ethnically matched controls [52]. This mutant *Nr2f2* protein has no transcriptional activity in a mouse model, eliminating synergistic transcriptional activation between NR2F2 and GATA4. Mutations that alter NR2F2 transcriptional activity with preserved repressor function were identified in patients with AVSD, TOF, aortic stenosis, CoA, and HLHS [53].

### 4.6. HAND Family

HAND1 and 2 are helix–loop–helix transcription factors that regulate, in a dose-dependent manner, the expansion of ventricular precursors [54]. In *Hand1* null mice, heart development is arrested at the heart looping stage of development [55]. *Hand1* conditional activation knock-in mice have increased expansion of the outer curvature of both ventricles but lack the interventricular groove and have a defect in formation of the septum. A mutation in *HAND2* has been associated with VSD, and HAND2 may have synergistic activation effects with GATA4 and NKX2-5 [56]. Many other transcription factors have also been shown to cause CHD when mutated, and the phenotypes resulting from the disruption of many of these are described in Table 1.

## 5. Signaling Pathways Underlying CHD

### 5.1. Nodal Signaling

An important signaling pathway in cardiovascular development is the Nodal signaling pathway known to regulating left–right patterning. Central to left–right patterning is Nodal expression that is restricted to the left side of the developing embryo. This initiates a signaling cascade that establishes left–right asymmetry. In CHD patients, there is evidence of the enrichment of heterozygous damaging de novo and loss-of-function mutations in *NODAL* [10]. *NODAL* mutations were identified in patients with TGA and a family history of CHD [49]. De novo CNVs affecting *NODAL* were also identified in a cohort of patients with conotruncal defects or HLHS [82]. Mutations in *ZIC3*, a transcription factor that functions upstream of *NODAL*, were identified in the aforementioned study, as well as in a study of CHD patients with HTX [83,84]. Mutations in several downstream targets of NODAL—*GDF1*, *CFC1*, *TDGF1*, *FOXH1*, and *SMAD*—were also identified in a cohort of CHD patients. Another downstream target of NODAL, *PITX2*, encodes a paired-like homeobox domain transcription factor that is a core effector of left–right patterning. A nonsense mutation identified in a family with endocardial cushion defect and Axenfeld–Rieger syndrome, which is associated with OFT defects, eliminates its transcriptional activity and synergistic transcriptional activation with NKX2-5 [85].

### 5.2. Notch Signaling

Signaling through the Notch pathway regulates cardiac cell fate and morphogenesis of cardiac chambers and valves [86]. Notch regulates EMT of the AV cushion progenitor cells which later contribute to the AV septum [87]. Rare deleterious variants in *NOTCH1* were identified in patients with strong family histories of disease [88]. *NOTCH1* mutations have previously been associated primarily with left-sided lesions, but a study of *NOTCH1* mutations in familial CHD identified individuals with right-sided and conotruncal defects [89]. While rare predicted loss-of-function and intronic variants in *NOTCH1* increase risk for left ventricular outflow tract defects [90], rare or likely pathogenic variants in *NOTCH1* have also been identified in a cohort of BAV patients requiring aortic root replacement [69], and de novo and rare variants were identified in patients with HLHS [91,92]. In addition, rare or novel protein-altering mutations in Notch pathway genes *NOTCH1*, *ARHGAP31*, *MAML1*, *SMARCA4*, *JARID2*, and *JAG1* were shown to co-segregate with disease in families with left ventricular outflow tract defects, and an enrichment of pathogenic variants in these genes in patients vs. controls was observed [93]. Heterozygous rare coding mutations in *MIB1*, which activates the Notch pathway through promoting ubiquitination, endocytosis, and activation of Notch ligands, were identified in a Han Chinese CHD cohort. Two of these mutations were shown to reduce function, resulting in less JAG1 ubiquitination and the induction of Notch [94]. This upstream effector *JAG1* is also associated with TOF [45]. Expression of Notch and its downstream targets are also reduced in mice with mutations in the Slit/Robo signaling pathway, resulting in membranous VSDs and BAV [95].

### 5.3. Wnt/β-Catenin Signaling

The Wnt/β-catenin pathway has an important role in many different aspects of heart development, including the regulation of cell proliferation in the SHF [96]. The recovery of candidate CHD genes in the Wnt pathway was observed in patients with bicuspid aortic valve (BAV) [37]. Enrichment for de novo variants in Wnt pathway genes has also been observed in CHD patients with neurodevelopmental defects, suggesting a shared genetic etiology [97]. Deletion of *Apc*, a negative regulator of canonical Wnt signaling, leads to ventricular hypoplasia in mice [98]. Context-dependent regulators of the Wnt pathway such as *Bcl9* and *Pygo* are also associated with cardiac defects, such as AVSD in mice or TOF in humans [99]. Canonical Wnt signaling is regulated by interactions between Dkk1/2, and mice that are double knockouts for *Dkk1* and *Dkk2* exhibit myocardial and epicardial hypoplasia, as well as VSD in later stages of development [100]. Non-canonical Wnt signaling also has been shown to activate the planar cell polarity (PCP) pathway, which coordinates processes such as chamber remodeling through actomyosin polarization and also regulates ciliogenesis [101,102,103]. Several core members of the PCP pathway were identified to cause cardiac defects in a mouse forward genetic screen [5]. Together with the finding of enrichment in other cilia-related genes, they indicate the importance of the PCP pathway in heart development and disease.

### 5.4. Bmp Signaling

Bmp signaling is required for specification and differentiation of the cardiac mesoderm and it regulates *Nkx2-5* expression through a negative feedback loop [96,104]. BMP4 deficiency can cause septal defects, defective endocardial cushion remodeling, and abnormal pulmonary valve formation, and common variants in *BMP4* are associated with CHD in a Han Chinese cohort [105]. Nonsynonymous variants in *SMAD6*, an inhibitor of Bmp signaling, have been identified in CHD patients [106]. *Furin* deletion targeted to endothelial cells in mice can reduce *Bmp4* and *Et1*, causing VSDs and valve malformations [107]. Also recovered were multiple de novo variants in *SMAD2* [108,109], which transduces Bmp signaling by regulating downstream target gene transcription [109]. De novo protein-truncating, splicing, and deleterious missense variants in *SMAD2* were identified in a cohort of CHD patients with a variety of defects including complex CHD with or without laterality defects and other congenital anomalies and late-onset vascular phenotype [110]. Mutations have also been recovered in *GALNT1*, a glycosyltransferase that can increase Bmp and Mapk signaling, causing aberrant valve formation due to increased cell proliferation in the outflow cushions [111]. Other studies suggest BMP10 plays a role in maintaining expression of NKX2-5 and other key cardiogenic factors to regulate cardiac growth [104]. *HIC2* encodes a transcriptional repressor that may regulate BMP10 in the FHF lineage specified by *NKX2-5* and *MESP1*. *HIC2* is impacted by the 22q11 deletion associated with DiGeorge syndrome [112].

### 5.5. Sonic Hedgehog (SHH) Signaling

**Figure 1:**
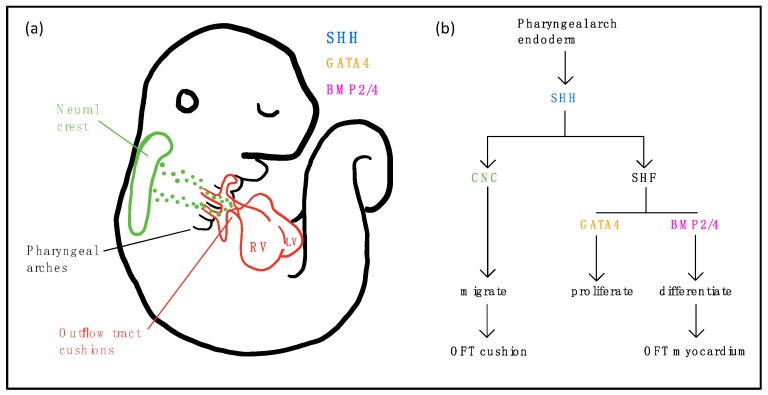
Diagram (**a**) and flowchart (**b**) illustrating the roles of Sonic Hedgehog (SHH) in OFT development. SHH (blue) is secreted from the pharyngeal arch endoderm. SHH signaling mediates migration and localization of cardiac neural crest (CNC) cells (green) to the outflow tract (OFT) endocardial cushions (red). SHH-receiving cells expressing GATA4 (orange) proliferate in the SHF, and those receiving signals from BMP2/4 (pink) differentiate into OFT myocardium.

SHH signaling has been shown to play an important role in the development of the SHF, outflow tract septation, and proper outflow tract alignment [113,114]. SHH is secreted from the pharyngeal endoderm, and ligand is received by SHF cells, maintaining proliferation of these progenitor cells (Figure 1) [113]. GATA4 was shown to be required for proliferation of SHH-receiving cells and subsequent OFT alignment, and *Gata4* mutations in mice cause DORV [115]. Signaling from BMP2 and BMP4 in the outflow tract myocardium, conversely, represses proliferation of SHH-receiving cells, with overexpression leading to premature differentiation of SHF cells and knockout resulting in embryonic lethality (Figure 1) [116]. SHH regulates development of SIX2+ progenitor cells, which contribute to the right ventricle, inflow tract, pulmonary trunk and ductus arteriosus [117]. Ablation of *Six2+* cells in mice was shown to result in severe CHD such as common arterial trunk. SHH is also required for migration of cardiac neural crest cells to the OFT cushion, with *SHH* mutations in mice resulting in neural crest cell death and mislocalization (Figure 1) [114]. Mutations in *Megf8* can cause TGA or other complex CHD associated with HTX [118]. While *Megf8* was previously proposed to regulate Tgfβ/Nodal signaling, a CRISPR screen recently identified *Megf8* as a negative regulator of SHH signaling [119]. Moreover, another negative SHH regulator identified in the same screen, *Mgrn1*, was also previously shown to cause HTX with CHD, with the CHD comprising TGA [120]. In fact, the role of SHH in human CHD has not been systematically examined, but the recovery of other regulators of SHH signaling among mutations causing CHD from a large scale mouse mutagenesis screen would suggest this pathway is likely to play an important role in human CHD [5].

### 5.6. Ras/Mapk Signaling

The Ras/Mapk pathway, which regulates proliferation, growth, and other cell processes, is also known to play important roles in CHD. Thus, disruption of the Ras/Mapk pathway results in a number of related disorders collectively termed RASopathies, the most common of which is Noonan syndrome. Noonan syndrome has the highest incidence of CHD, particularly pulmonary stenosis, among RASopathy patients [3]. *PTPN11*, which encodes an upstream regulator of the Ras pathway, is well known to cause Noonan syndrome and is enriched for de novo mutations in a cohort of syndromic CHD patients [16]. A de novo mutation in *MRAS*, which contributes to ERK activation and downstream Mapk signaling, was identified in a patient with Noonan syndrome and cardiac hypertrophy [121]. Noonan syndrome patients were also identified with heterozygous de novo and inherited mutations in *A2ML1*, which may act upstream of the Ras signaling pathways. However, in cell lines, expression of A2ML1 did not activate the Ras/Mapk pathway [122].

### 5.7. Vegf Signaling

The Vegf signaling pathway is required for formation of the AV endocardial cushions and their morphogenesis into AV valves [123]. In a cohort of TOF patients, predicted damaging variants were identified in the Vegf-related genes *FLT4*, *KDR*, *VEGFA*, *FGD5*, *BCAR1*, *IQGAP*, *FOXO1*, and *PRDM1*. These variants are associated with absent pulmonary valve and right aortic arch [67]. In a cohort of patients with Down syndrome and AVSD, variants with the highest probability of being damaging in cases compared to Down syndrome patients without cardiac defects were in the VEGF-A pathway genes *COL6A1*, *COL6A2*, *CRELD1*, *FBLN2*, *FRZB*, and *GATA5* [123]. Signaling pathway genes that have been shown to cause CHD when mutated in mice and humans, as well as their resulting phenotypes, are described in Table 2.

## 6. Myofilament and Extracellular Matrix Proteins

Proteins that compose the sarcomere and extracellular matrix are essential for proper structure and function of cardiac muscle. Mutations in *ACTC1*, *DCHS1*, *TTN*, *ELN*, *MYH6*, *MYH7*, and *MYH11* are known to cause cardiac defects [3]. *MYH6* mutations have been associated with atrial septal defects (ASD) and recently were shown to be significantly associated with CoA in a GWAS study of an Icelandic population [124]. TPM1, an essential component of the sarcomere, has been associated with cardiomyopathy [125]. Mutations in the cytoskeletal protein *ACTC1* cause ASD that is thought to arise from cardiomyocyte apoptosis [126,127]. The actin-binding protein NEXN has also been associated with ASD [32]. Genes that regulate splicing of essential cardiac genes are also known to cause CHD. The splicing factor *RBM20* regulates alternative splicing of genes associated with diastolic function and ion transport, as well as sarcomere assembly, particularly *TTN* where greater RBM20 expression is associated with the expression of shorter isoforms of TTN [128]. In mice, mutations in *Rbm20* result in dilated cardiomyopathy (DCM) with similar severity to *Ttn* mutations, and arrhythmia that is more severe than *Ttn* mutations, indicating a role for other *Rbm20* targets in disease [129].

Cells must be able to respond and adhere to other cells and the extracellular matrix to maintain structure and transduce intracellular signaling. In mice, deficiency in the matrix protein *Ccn1*, which regulates cell adhesion and migration, proliferation, survival, and differentiation, results in severe AVSD [130]. Mutations in *BVES,* a cell adhesion protein, were identified in TOF patients. One *Bves* mutation was shown to alter transcriptional activity in a cell based assay [131]. *Pcdha9*, encoding a protocadherin cell adhesion protein, was shown to have an essential role in valvular morphogenesis, as *Pcdha9* mutation can contribute to the aortic hypoplasia/atresia in HLHS and also can cause bicuspid aortic valve (BAV) [79].

## 7. Chromatin Modifiers

Chromatin modifiers regulate the epigenetic marks that control DNA accessibility and transcriptional activity. Disruption of these processes can interfere with transcriptional programs important for orchestrating events in cardiovascular development. Chromatin modifiers were found to be enriched among genes with de novo mutations in a CHD cohort with diverse phenotypes including LVOTO, conotruncal defects, and HTX [10]. Several genes involved in the regulation of active H3K4me/inactive H3K27me histone marks were identified. *KMT2D* encodes one of these histone modifiers and is associated with Kabuki Syndrome with CoA, ASDs, and VSDs [152]. Mutations have also been recovered in *CASZ1* encoding a zinc finger transcription factor that interacts with histones and is essential for cardiogenesis. A *CASZ1* mutation associated with reduction in transcriptional activity caused VSD as a completely penetrant autosomal dominant trait [153].

The HDAC repressor complex plays a key role in many developmental processes, and several proteins that are associated with this complex are associated with CHD [154]. Thus, variants in *SMYD4*, a protein which interacts with HDAC1 and can modulate histone acetylation [155], were identified in patients with DORV and TOF. Genes regulating chromatin were also identified in *Smarca4* and *Prdm1* in a mouse forward genetic screen for CHD [5]. Another mutant recovered from the same screen harbored a CHD-causing mutation in *Sap130*, a Sin3A associated protein that is also part of the HDAC repressor complex. Mutation in *Sap130* was shown to mediate left ventricular hypoplasia [156]. Double homozygous *Pcdha9* and *Sap130* mutations were shown to cause HLHS, with the *Pcdha9* mutation found to drive the aortic valve phenotype associated with HLHS [79].

## 8. The Role of Cilia and Cilia-Transduced Cell Signaling During Cardiogenesis

**Figure 2:**
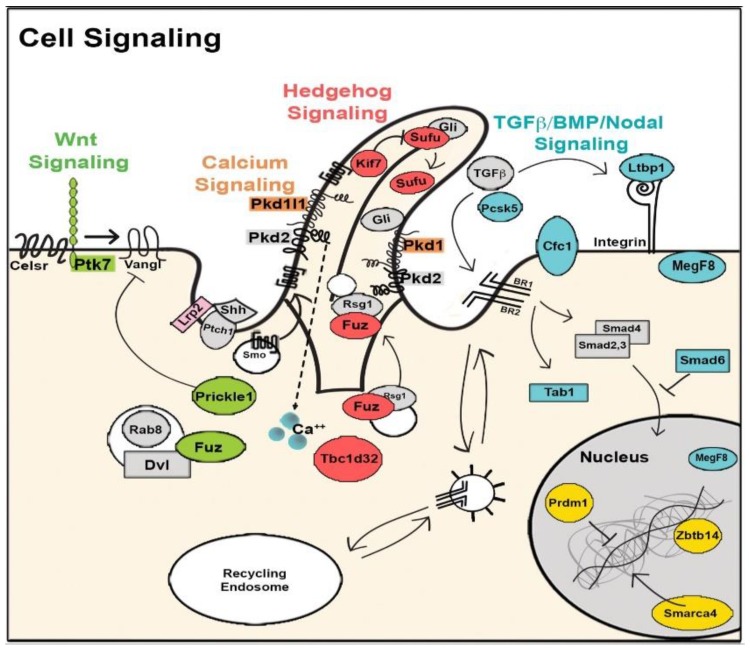
Diagram illustrating the biological context of cilia in signaling pathways involved in heart development. Highlighting denotes recovery from the CHD screen. R, receptor. Adapted from [5].

The cilium is an organelle that protrudes from the cell surface and can be motile or nonmotile. Motile cilia are involved in cell motility and the generation of extracellular fluid flow, such as in mediating mucociliary clearance in the airway or cerebral spinal fluid flow in the brain. During early embryonic development, motile cilia in the embryonic node generate flow responsible for creating a gradient of signaling molecules, such as NODAL, that establishes left–right patterning. This is essential for normal cardiac morphogenesis, as disruption of left–right patterning causing HTX is associated with some of the most complex forms of CHD. Nonmotile cilia, known as primary cilia, can function as cell signaling transducers or serve as mechanosensors. Cilia and cilia-transduced cell signaling can modulate planar cell polarity and affect cytoskeletal organization involved in the regulation of EMT. This is essential for emergence of neural crest cells from the neural tube, epicardially-derived cells from the epicardium, and development of the cardiac cushion mesenchyme from endocardial EMT [157]. In addition, cell signaling pathways known to play essential roles in heart development, such as Wnt, Tgfb/Bmp, and SHH are all cilia-transduced (Figure 2) [158].

**Figure 3:**
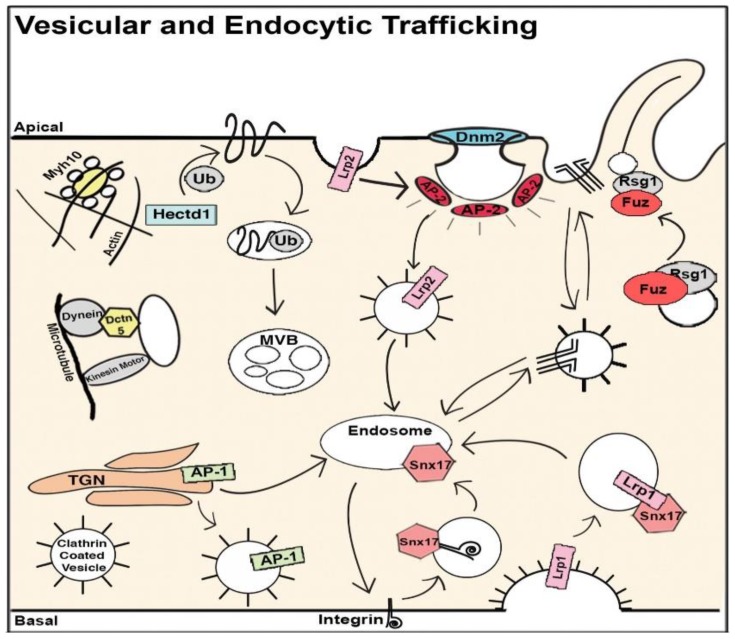
Diagram illustrating the genes recovered from the CHD screen that are required for ciliogenesis. IFT, intraflagellar transport; TGN, trans-Golgi network. Adapted from [5].

**Figure 4:**
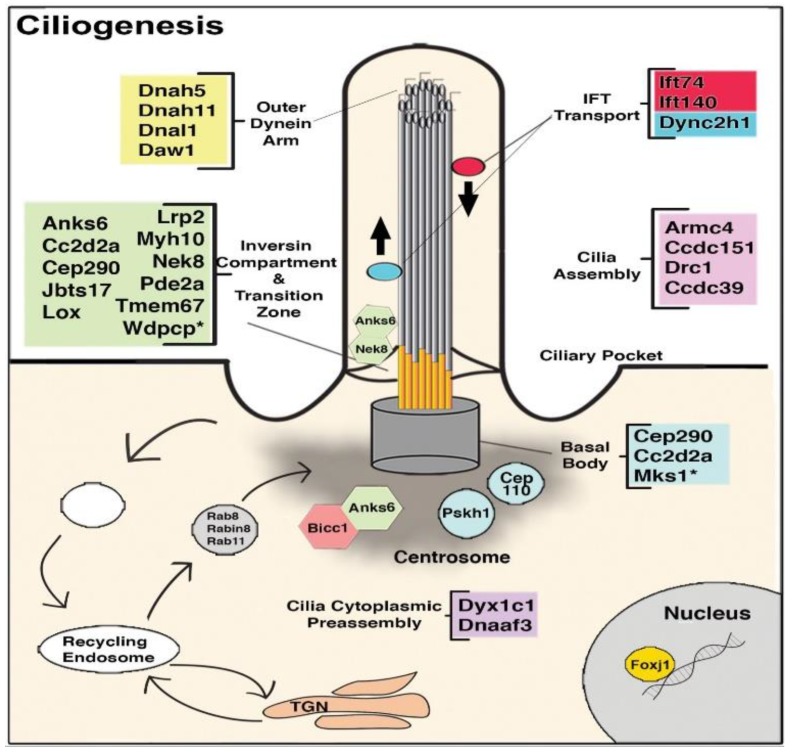
Diagram illustrating the biological context of ciliary genes in vesicular and endocytic trafficking. Highlighting denotes recovery from the CHD screen. AP, adaptor protein complex; MVB, multivesicular body; Ub, ubiquitination. Adapted from [5].

**Figure 5:**
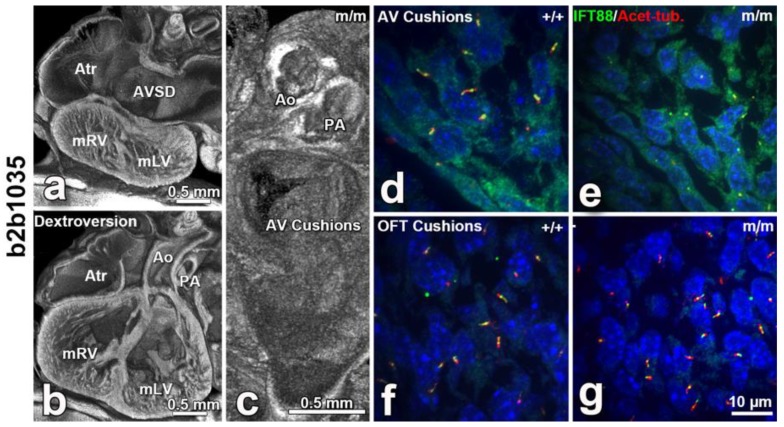
*Cc2d2a*-mutant mouse (line b2b1035) exhibits dextrocardia with ventricular inversion (dextroversion) (**b**), and AVSD (**a**) with malformed atrioventricular cushions (**c**), but normal outflow cushions. Atr, atrium; mLV, morphologic left ventricle; m/m, Cc2d2a-mutant mouse; mRV, morphologic right ventricle. Confocal imaging of E12.5 *Cc2d2a*-mutant mouse (m/m) versus wild-type (+/+) embryo sections showed no cilia in the atrioventricular cushion (**d**,**e**), but normal ciliation in the outflow cushion (OFT cushion) (**f**,**g**). Adapted from [5].

A central role for cilia in CHD pathogenesis was discovered via the use of forward genetics in mice with ENU mutagenesis to recover mutations causing CHD. Cardiovascular phenotype was assessed using fetal echocardiography, a noninvasive high-throughput phenotyping method that is also highly sensitive for the detection of CHD and allowed the screening of 100,000 fetal mice. While the screen was entirely phenotype-driven, surprisingly 50% of the mutations recovered causing CHD were cilia related. This encompassed mutations in 30 genes related to cilia and ciliogenesis (Figure 3). Additionally, the screen also recovered many genes involved in cilia-transduced cell signaling (Figure 2) and in vesicular trafficking (Figure 4), a cell process critical for ciliogenesis and cilia-transduced cell signaling [5]. A separate mouse screen also identified mutations in *Dnah11*, an axonemal protein, and *Mks1*, a basal body protein, to be associated with CHD [159]. Mutations in these same genes were also recovered in the large scale fetal mouse CHD screen. The ciliary gene *Ift88* is an intraflagellar transport protein required for cilia formation, and *Ift88* null mutant mice exhibited OFT defects. [160,161]. Cilia have also been shown to play a role in aortic valve disease, such as BAV [162]. Defects in development of the AV cushions in *Cc2d2a* mutant mice were associated with loss of cilia from the AV cushions (Figure 5). In human studies, an enrichment of ciliary genes was observed among genes with damaging recessive variants in a CHD cohort [10]. We note analysis of the early mouse embryos has revealed primary cilia in the endocardium of the atria, the endocardial cushions, and the cushion mesenchyme, as well as in the epicardium [163]. Ciliary defects have only recently been identified as a cause of CHD, and their role in the developmental processes of the heart and the contribution to CHD pathogenesis warrants further studies.

## 9. Maternal Effects

Maternal genetics and behavior should also continue to be studied in relation to their effects on fetal cardiac development, as changes in the fetal environment have been associated with CHD. Congenital heart disease has been associated with maternal smoking, parental age, and maternal fertility and nonfertility medications [164], as well as maternal obesity [165], maternal alcohol consumption [166], and maternal viral infection [167]. CHD pathogenesis in these cases has been attributed to impacts on placental development [168], overactive maternal immune responses [169], and deficiency of folic acid, which is essential for fetal growth and development [170,171].

## 10. Future Directions

CHD is a heterogeneous disease with complex genetics underlying its pathogenesis. While a large body of evidence points to CHD being genetically heterogeneous, there may be a central role for cilia and chromatin modifiers in driving the complex genetics of CHD. However, the molecular mechanisms driving CHD pathogenesis are still not well understood. Mouse models with genetic mutations causing CHD is an invaluable resource for further mechanistic studies. Findings from these animal models may help guide assessments and validation of the role in disease of various sequence variants recovered from patients with CHD. Such pairing of animal studies with clinical findings may give novel insights not only into molecular mechanisms of human CHD, but the animal models generated may provide the means to develop therapies that may have improve outcome for patients with CHD. Similarly, large-scale studies of human cohorts will continue to reveal novel variants that are relevant to disease and their effects on phenotype and outcome. Stratification of analyses based on specific phenotypes, patient outcomes, and variant predictions will further reveal the genetic architecture underlying CHD. In addition, greater focus on common and non-coding variants can help uncover the role that these variants play in disease, particularly in the context of known rare and deleterious variants. Further investigation into epigenetics and the effects of maternal genetics will also be needed to obtain a full picture of the risk factors contributing to the penetrance and pathogenesis of CHD.

## Figures and Tables

**Table 1:** Transcription factors associated with congenital heart disease (CHD) and their phenotypes in patients and mice.

Gene	Human Phenotype	Mouse Phenotype	References
CITED2	AS, PS, SIT, Dextrocardia, TGA, TOF, RVOTO, TAPVR, ASD, VSD	DORV, PTA, OA, AA, PAA anomaly, ASD, VSD	[20,57,58,59]
CREBBP	Rubinstein-Taybi syndrome	CHD	[60,61]
EP300	Rubinstein-Taybi syndrome	Hypotrabeculation, Thin myocardium, ASD, VSD	[60,62]
ETS1	DORV, HLHS, ASD, VSD	ASD, VSD	[20,63]
FOXC1	HLHS, OA, PA, PAH, PDA, Bilateral SVC, VSD, Axenfeld–Rieger syndrome, ASD	Aortic arch defects, IAA, Inflow tract defects, OFT defects, RV defects, Semilunar valve defects, VSD	[20,43,57,61]
FOXC2	HLHS, TOF, OA, PA, PDA, PAH, TAPVR, Bilateral SVC, ASD, VSD	Aortic arch defects, IAA, Inflow tract defects, OFT defects, PTA, RV defects, Semilunar valve defects, VSD	[20,43,57,61]
FOXH1	TOF, TGA, HTX, VSD	Disorganized myocardium, OFT defects, RV defects	[43,60,64,65,66]
FOXJ1	CHD	Complex CHD with HTX	[5,44]
FOXO1	TOF	Endocardial cushion defects, Reduced trabeculations	[43,67]
FOXP1	CHD	Defects in ventricular/OFT septation, valve formation, myocardial proliferation	[43,60]
GATA4	Dextrocardia, AVSD, DORV, TOF, BAV, CoA, AR, PAPVR, PDA, PS, ASD, VSD	Acardia, Cardia bifida, AVSD, DORV, PTA, ASD, VSD	[20,57,63,64]
GATA5	AVSD, DORV, LVNC, BAV, CoA	BAV	[20,68,69,70]
GATA6	AVSD, TOF, PDA, PTA, PS, ASD, VSD	Acardia, AVSD, DORV, PTA, IAA, PAA anomaly, ASD, VSD	[33,57,63,64]
HAND1	AVSD, DORV, HLHS, HLV, HRV, ASD, VSD	Arrest at looping stage, VSD and hypoplastic AV valves, Absent ventricular septum and thin compact myocardium	[54,55,57,71,72,73]
HAND2	TOF, LVNC, VSD	DORV, HRV, PAA anomaly, PS, VSD	[20,59,64,70,74]
JARID2	Left-sided lesions	DORV, Hypertrabeculation, Myocardial defects, Noncompaction, VSD	[20,62,75]
MSX1	BAV, CoA	DORV, TOF, PTA, Hypoplastic valves, VSD	[20,37,59]
NFATC1	TOF, LVNC, BAV, CoA, TA, VSD	Absent valves, Blunting of AV/OFT valves, VSD	[20,37,57,70,76]
NKX2-5	ASD, AVSD, BAV, CoA, Dextrocardia, DORV, Ebstein’s anomaly, HTX, HLHS, IAA, LVNC, Mitral valve anomalies, PA, PAPVR, PDA, PS, SVAS, TA, TAPVR, TGA, TOF, PTA, VSD	AVSD, Looping defect, ASD, VSD	[25,57,61,63,64,74]
NR1D2	AVSD	AVSD	[50]
NR2F2	AVSD, DORV with VSD	Hypoplastic atria, Ventricularized atria	[51,52,53,74]
RBPJ	HLHS	Defective EMT, Hypoplastic endocardial cushions, Impaired trabeculation, VSD	[20,77,78,79]
RFX3	PTA	HTX	[80,81]
SMAD6	HLHS, AS, BAV, CoA	DORV, TGA, PTA, IAA, RAA, Hypoplastic pulmonary artery, Aortic valve dysplasia, Hyperplastic valves, VSD	[5,24,64,79]
TBX1	DORV, TOF, IAA, PTA, VSD, DiGeorge syndrome, Velocardiofacial syndrome	AVSD, DORV, TGA, TOF, PTA, PAA anomaly, VSD	[20,57,60,63,64]
TBX2	CHD	DORV, Hypoplastic endocardial cushions, PAA anomaly	[20,60]
TBX20	DORV, HLV, LVNC, DCM, CoA, MS, PDA, ASD, VSD	AVSD, DORV, PTA, Hypoplastic right heart, ASD, VSD	[20,59,63,70,71]
TBX3	Ulnar-Mammary syndrome	DORV, TGA, PAA anomaly, VSD	[61,80]
TBX5	AVSD, TOF, BAV, CoA, ASD, VSD, Holt-Oram syndrome	ASD, VSD	[37,45,57,61,63]
ZFPM2	AVSD, DORV, TOF, VSD	Alignment defects, Coronary artery defects, OA, PS, TA, ASD, VSD	[20,50,64,74,81]

AA, aortic atresia; AR, aortic regurgitation; AS, aortic stenosis; ASD, atrial septal defect; AV, atrioventricular; AVSD, atrioventricular septal defect; BAV, bicuspid aortic valve; CoA, Coarctation of the aorta; DCM, dilated cardiomyopathy; DORV, double outlet right ventricle; EMT, epithelial-to-mesenchymal transition; HLHS, hypoplastic left heart syndrome; HLV, hypoplastic left ventricle; HRV, hypoplastic right ventricle; HTX, heterotaxy; IAA, interrupted aortic arch; LVNC, left ventricular noncompaction; MS, mitral stenosis; OA, overriding aorta; OFT, outflow tract; PA, pulmonary atresia; PAA, pharyngeal arch artery; PAH, pulmonary artery hypoplasia; PAPVR, partial anomalous pulmonary venous return; PDA, patent ductus arteriosus; PTA, persistent truncus arteriosus; RAA, right-sided aortic arch; RV, right ventricle; RVOTO, right ventricular outflow tract obstruction; SIT, situs inversus totalis; SVAS, supravalvular aortic stenosis; SVC, superior vena cava; TA, tricuspid atresia; TAPVR, total anomalous pulmonary venous return; TGA, transposition of the great arteries; TOF, tetralogy of Fallot; VSD, ventricular septal defect.

**Table 2:** Cell signaling genes associated with CHD and their phenotypes in patients and mice.

Gene	Human Phenotype	Mouse Phenotype	References
**Notch Signaling**			
ADAM17	AVSD	CHD	[50]
HES1	TGA	OA, PAA anomalies, VSD	[59,78,81]
HEY2	AVSD	TOF, HRV, OA, TA, PS, Thickened mitral valve, ASD, VSD	[20,78,132]
JAG1	Aortic dextroposition, TOF, BAV, CoA, PS, VSD, Alagille syndrome	DORV, PTA, TOF, IAA, OA, AAAD, PS, Thickened or calcified valves, ASD, VSD	[20,37,57,61,74,78,133]
NOTCH1	HTX, AVSD, TOF, HLHS, LVNC, BAV, CoA, AS, MS, VSD	Aberrant trabeculation, DORV, HRV, Hypoplastic endocardial cushions, Impaired EMT, IAA, PAA anomalies, PS, PTA, TA, Valve defects, ASD, VSD	[10,20,24,37,50,61,63,64,66,71,77,78,134,135]
NOTCH2	AVSD, TOF, BAV, CoA, PS, Alagille syndrome	PS, Reduced compact myocardium, ASD, VSD	[20,37,50,61,68,78,133]
**WNT/β-Catenin Signaling**			
APC	BAV, CoA	Ventricular hyperplasia	[37,98]
BCL9	CHD	Septal defects, Valve defects	[99,108]
DCHS1	LVNC, Mitral valve prolapse	Prolapsed, thickened mitral leaflets	[70,136]
DVL1	LVNC, PDA	CHD	[60,64]
EDN1	TOF	DORV, PTA, PAA anomaly, VSD	[20,74,137]
PCDHA9	HLHS	HLHS, BAV, Aortic hypoplasia/stenosis	[79]
**TGF-β/BMP/Nodal Signaling**			
ACVR1	HTX, AVSD, DORV, TGA, Left-sided lesions, ASD	PTA, PAA anomaly, ASD, VSD	[20,71,75,138]
ACVR2B	HTX, Dextrocardia, AVSD, DORV, TGA, HLHS, LSVC, PS, Venous anomaly	HTX, TGA, DORV, AA	[59,61,64,66,139]
BMPR1A	AVSD	Hypoplastic endocardial cushion, Impaired EMT, PTA, ASD, VSD	[20,77,140,141]
BMPR2	AVSD, PDA, PAPVR, ASD, VSD	Absent OFT valves, AV cushion defect, DORV, PTA, IAA, OA, Thickened valve leaflets, ASD, VSD	[59,61,91,138]
GDF1	HTX, AVSD, DORV, TGA, TOF	HTX, DORV, TGA, TOF	[10,59,63,71,142]
SMAD6	HLHS, AS, BAV, CoA	DORV, TGA, PTA, IAA, RAA, Hypoplastic pulmonary artery, Aortic valve dysplasia, Hyperplastic valves, VSD	[5,20,24,64,79,134]
TGFB2	VSD, Loeys-Dietz syndrome	DORV, DILV, PTA, Hypoplastic endocardial cushions, Hypoplastic aortic arch, OA, PAA anomaly, TAAD, BAV, Abnormal AV valves, Hyperplastic valves, VSD	[20,59,63,77,81,143]
TGFB3	Loeys-Dietz syndrome	VSD	[20,63]
TGFBR1	BAV, Myxomatous mitral valve, TAAD, Loeys-Dietz syndrome, Marfan syndrome	Hypoplastic endocardial cushions, PTA, PAA anomaly, VSD	[20,61,63,66,69,138,144,145]
TGFBR2	HTX, Mitral valve prolapse, Myxomatous mitral valve, TAAD, Loeys-Dietz syndrome, Marfan syndrome	DORV, PTA, OA, PAA anomaly, Tricuspid valve defect, ASD, VSD	[20,61,63,66,138,142,144,145,146,147]
**RAS/MAPK Signaling**			
BRAF	Cardiofaciocutaneous syndrome, Costello syndrome, LEOPARD syndrome, Noonan syndrome	Cardiac defects modeling cardiofaciocutaneous syndrome	[61,63,68,133,148,149]
PTPN11	AVSD, CoA, AS, PS, Cardiofaciocutaneous syndrome, Costello syndrome, LEOPARD syndrome, Noonan syndrome	AVSD, DORV, PTA, Valve defects, ASD, VSD	[20,60,61,63,68,134,149,150,151]
SOS1	AVSD, PS, Cardiofaciocutaneous syndrome, Costello syndrome, LEOPARD syndrome, Noonan syndrome	Valve defects	[60,61,63,64,68,108,134,144,148]
**VEGF Signaling**			
ETS1	DORV, HLHS, ASD, VSD	ASD, VSD	[20,57,63,81]
VEGFA	TOF, PDA, PTA, AS, BAV, CoA, IAA, VSD	EMT defects, DORV, TOF, Blunted AV valves, VSD	[20,24,64,71]

AA, aortic atresia; AAAD, aortic arch artery defect; AS, aortic stenosis; ASD, atrial septal defect; AV, atrioventricular; AVSD, atrioventricular septal defect; BAV, bicuspid aortic valve; CoA, Coarctation of the aorta; DILV, double inlet left ventricle; DORV, double outlet right ventricle; EMT, epithelial-to-mesenchymal transition; HLHS, hypoplastic left heart syndrome; HRV, hypoplastic right ventricle; HTX, heterotaxy; IAA, interrupted aortic arch; LSVC, left superior vena cava; LVNC, left ventricular noncompaction; LVOTO, left ventricular outflow tract obstruction; OA, overriding aorta; OFT, outflow tract; PAA, pharyngeal arch artery; PAPVR, partial anomalous pulmonary venous return; PDA, patent ductus arteriosus; PS, pulmonary stenosis; PTA, persistent truncus arteriosus; RAA, right-sided aortic arch; TA, tricuspid atresia; TAAD, thoracic aortic aneurysm and dissection; TAPVR, total anomalous pulmonary venous return; TGA, transposition of the great arteries; TOF, tetralogy of Fallot; VSD, ventricular septal defect.
